# Antiparasitic Potential of Bioactive Compounds From *Punica granatum*: Preventive and Therapeutic Effects

**DOI:** 10.1155/japr/4715270

**Published:** 2026-06-26

**Authors:** José Roberto Vargas-Villanueva, Filiberto Gutiérrez-Gutiérrez, Verónica Yadira Ochoa-Maganda, Sendar Daniel Nery-Flores, Lissethe Palomo-Ligas

**Affiliations:** ^1^ Facultad de Ciencias Químicas, Universidad Autonoma de Coahuila, Unidad Saltillo, Saltillo, Coahuila, Mexico; ^2^ Departamento de Farmacobiología, Centro Universitario de Ciencias Exactas e Ingenierías, Universidad de Guadalajara, Guadalajara, Jalisco, Mexico, udg.mx; ^3^ Departamento de Química Aplicada, Universidad Tecnológica de Jalisco, Guadalajara, Jalisco, Mexico

**Keywords:** natural products, neglected tropical diseases, parasites, pomegranate

## Abstract

Diseases caused by parasites are a global public health problem. Due to their heterogeneity and diversity, they can affect various hosts, and some diseases are considered zoonotic. Therapeutic control in parasitic diseases has lost its effectiveness over the last decades. The factors associated with therapeutic failure include drug resistance, nonadherence to treatment due to the presence of adverse effects, and reinfections due to inadequate sanitary conditions. Therefore, a strategy to search for new treatment alternatives is the use of natural compounds. The pomegranate is a plant that has multiple biological activities, such as antioxidant, anti‐inflammatory, and antimicrobial. It is highlighted that the use of aqueous and alcoholic extracts of pomegranate, mainly from the peel, has greater activity against clinically relevant pathogenic parasites. The use of *Punica granatum* as a preventive, monotherapy, or adjuvant agent demonstrates its protective and restorative capacity in the damage produced by various parasitoses. These effects are mainly attributable to phenolic compounds, principally ellagitannins and ellagic acids. Among these molecules, punicalagin, punicalin, luteolin, epigallocatechin gallate, gallic acid, chlorogenic acid, catechin, and quercetin stand out. The identification and study of these candidates is a promising alternative to complement the arsenal of available antiparasitic drugs. In this research, the antiparasitic activity of pomegranate is summarized.

## 1. Introduction

Parasites are eukaryotic pathogens that cause several diseases with significant impacts on humans, including protozoa, helminths, and arthropods [1]. Intestinal parasitic infections alone affect approximately 3.5 billion individuals worldwide, leading to over 200,000 deaths annually. These infections disproportionately impact impoverished populations, perpetuating cycles of poverty and hindering socioeconomic development [2, 3].

The emergence of drug‐resistant parasite strains and the adverse effects associated with existing antiparasitic medications necessitate the exploration of novel therapeutic agents. Natural products have garnered attention for their potential in this regard [4].

The pomegranate (PG) (*Punica granatum* L.), belonging to the Punicaceae family, is a fruit‐bearing tree native to Asia and now cultivated worldwide [5]. Various parts of the PG, including the peel, seeds, and flowers, have been traditionally used for their medicinal properties [6]. Several studies have demonstrated the fruit′s antimicrobial, anti‐inflammatory, cardioprotective, and antioxidant activities, among others [7].

In this review, the antiparasitic potential of PG is summarized, highlighting its applications as a preventive measure, monotherapy, or adjuvant to conventional treatments. This therapeutic potential is supported by the rich phytochemical composition of *P. granatum*, which includes polyphenols (particularly ellagitannins such as punicalagin and punicalin), phenolic acids (gallic, ellagic, chlorogenic, caffeic, and syringic acids), flavonoids (catechin, quercetin, luteolin derivatives, and naringenin), as well as sterols, triterpenes, saponins, alkaloids, anthocyanins, glycosides, and quinones [8, 9]. These metabolites have demonstrated antiparasitic effects in both in vitro and in vivo studies, including direct activity against parasite growth and survival, additive or synergistic interactions with conventional drugs, and protective effects in infected animal models through antioxidant, anti‐inflammatory, and immunomodulatory mechanisms. Understanding the scope of PG′s efficacy against parasitic infections could provide a basis for developing alternative therapeutic strategies while also increasing the evidence and interest in its potential applications, particularly in resource‐limited settings where these diseases are most prevalent.

## 2. Phytochemistry of *P. granatum* L.

### 2.1. Ethnobotanical Characteristics of *P. granatum*

**Figure 1:**
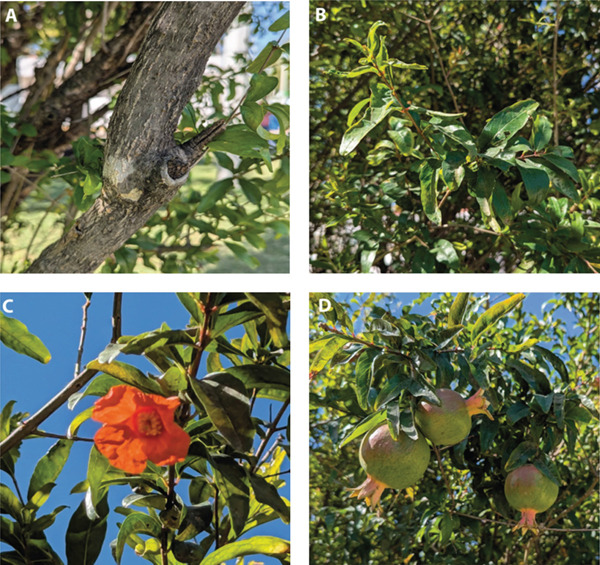
Components of the pomegranate tree: (A) stem bark, (B) leaves, (C) flower, and (D) fruit.

*P. granatum* is a small, highly branched, thorny, deciduous shrub or tree that can reach a height of 5–8 m. It is distributed in temperate and subtropical areas with Mediterranean climates. The species is quite tolerant to drought and moderate low temperatures [10]. The trunk is straight with cracked bark, which is rich in tannins, proanthocyanidins, anthocyanins, and terpenoids [11–15] (Figure 1A). This bark has been used ethnopharmacologically to treat diarrhea [16] and parasitic diseases such as malaria [17] and schistosomiasis [18]. The leaves are simple, measuring 1.5–4 cm in length and 0.8–2 cm in width, oblong with slightly lanceolate shapes, glossy on the upper side, and green (Figure 1B). Traditionally, the leaves have been noted for their antibacterial and antibiotic properties [19, 20], as well as their antidandruff and antilice activities [21]. They are rich in alkaloids, flavonoids, and tannins [14, 19, 20, 22].

The flowers of *P. granatum* are 3–4 cm in diameter, solitary, and hermaphroditic. The petals, measuring 2–2.5 cm by 1–1.5 cm, are obovate, wrinkled, and bright red (Figure 1C). These flowers usually bloom in summer and autumn. Among their traditional uses, the flowers are recognized for their antimicrobial [23, 24], antiamebic, and anthelmintic activities [25, 26]. Additionally, they are rich in bioactive components such as tannins, terpenes, terpenoids, flavonoids, and organic oils [14, 19, 24]. The fruit is a globular berry, 5–12 cm in size, leathery, reddish or yellow‐reddish in color, and crowned by the remnants of calyx segments (Figure 1D) [10]. The juice of the fruit is reddish and sweet, containing large quantities of antioxidants, especially polyphenolics such as punicalagin and punicalin [12, 27–30]. The juice also exhibits antileishmanial effects [27]. The seeds measure 12–15 mm by 5–7 mm, covered by red‐yellow arils, and have antimicrobial [24, 31], anthelmintic [32, 33], and antiamoebic properties [25]. They are also rich in anthocyanins, tannins, fatty acids, flavonoids, ligand compounds, sterols, and some volatile organic oils [14, 34–37]. Finally, the peel of *P. granatum* is a hard pericarp ranging from emerald red to orange–green when ripe, constituting over 40% of the fruit′s weight [10, 36]. Ethnopharmacologically, peel extracts are commonly used to treat diarrhea and have antiparasitic, anthelmintic, and antiprotozoal properties [37–41]. The peel of *P. granatum* contains alkaloids, anthocyanins, tannins, flavonoids, phenolics, proanthocyanidins, sterols, terpenes, and xanthonoids in PG peels [7, 15, 29, 42, 43].

#### 2.1.1. Phytochemical Extraction Methods (Traditional and Novel) for Obtaining Antiparasitic Compounds From *P. granatum*

The efficiency of extracting secondary metabolites with antiparasitic activity from *P. granatum* (peels, leaves, roots, seeds, and flowers) critically depends on the employed method [44]. In general, extraction methods can be classified into traditional ones, which use simple and well‐known systems for compound extraction, and novel ones, which employ advanced and efficient techniques, often “green” or sustainable, designed to separate active compounds by maximizing yield and quality while drastically reducing processing time, temperature, and the use of toxic organic solvents [44, 45].

##### 2.1.1.1. Traditional Methods

Cold maceration: It consists of immersing the dried and powdered plant material in a solvent at room temperature for relatively long periods, with occasional or continuous stirring. The advantages of this method include its simplicity, as it requires no specialized equipment; however, it demands a long extraction time and high solvent consumption [45, 46].

For PG, maceration with water or solvent mixtures such as methanol, ethanol, and acetone has been used. The type of solvent or solvent mixture is very important during the maceration process, as it allows increased extraction yields from PG, especially for extracting tannins and flavonoids [7, 47].

Decoction: In this method, the plant material is boiled in the solvent, usually water, for a few minutes. This process promotes the disruption of the plant cell wall, increasing the extraction rate; however, its main disadvantage is that it can easily degrade thermosensitive compounds [46, 48]. This method is used to extract polysaccharides and hydrolyzable tannins from PG peel, allowing rapid and reproducible extraction processes [49].

Percolation: In this method, the solvent flows continuously through a column packed with plant material at a controlled rate. It allows more efficient extraction than simple maceration in a shorter time; however, it requires large volumes of solvents [45, 50]. In *P. granatum*, percolation has been used with solvents such as distilled water, acetone, methanol, and ethanol, allowing the extraction of components of various polarities. Additionally, extraction complexes with cyclodextrins have been used, improving polyphenol extraction by up to 20% [51, 52].

Soxhlet extraction: This is a continuous reflux method that uses a hot solvent that repeatedly circulates through the plant material. Its main advantage is its high yield; however, thermolabile compounds are especially sensitive to degradation, and it also requires high energy consumption [7, 44]. For PG, a wide variety of solvents have been employed, such as water, ether, hexane, chloroform, benzene, methanol, acetonitrile, and ethanol, to extract fatty acids (from seeds) and flavonoids (from leaves and peels), showing a good capacity to extract phenolic compounds (196 mg GAE/g), in particular gallic acid with up to 59.8 ± 0.6 mg GAE/g [52, 53].

##### 2.1.1.2. Novel Methods (Green Technologies)

Ultrasound‐assisted extraction: It uses ultrasonic waves that generate acoustic cavitation, breaking cell walls and facilitating the release of compounds. The advantages of this method are improved extraction efficiency, reduced time, and better thermal stability of the compounds [44, 46].

It has been shown that in PG extracts, under conditions of 15–40 min; temperature 30°C–50°C; power 75–500 W; solvents such as ethanol, methanol, water, and acetone; and a solid‐to‐liquid ratio of 1:10–1:30, the ultrasound extraction method increases yield and reduces processing time; for example, it increases the yield of punicalagins and ellagic acid in peels by 30%–50% compared to maceration, enhancing antioxidant activity and total phenolic content [7, 47].

Microwave‐assisted extraction: It takes advantage of dielectric heating that causes intracellular evaporation and tissue disruption in minutes, with high extraction efficiencies, short times, and minimal temperature increase [7, 47].

The extraction of compounds from PG is a rapid, efficient green technology that optimizes the recovery of phenolic compounds, flavonoids, and antioxidants. Optimal conditions typically involve power 400–800 W; time 2–10 min; temperature 50°C–80°C; and polar solvents such as ethanol, water, methanol, and acetone, yielding up to 56% efficiency [54, 55].

Supercritical fluid extraction: It uses CO_2_ under supercritical conditions as a solvent, with the possibility of adding cosolvents to increase polarity. In general, it is nontoxic and nonflammable, allowing compounds to remain stable, with a significant improvement in extraction efficiency and bioactive compound yield [44, 47].

In the case of PG, this method is mainly used for the extraction of volatile compounds and essential oils, primarily from PG peel, requiring less time compared to the traditional hydrodistillation method, and less energy to operate, making it more sustainable. Common operating parameters are 40°C–60°C, 150–300 bar, CO_2_ flow rate 2–5 L/min, and extraction time 1–2 h. Ellagic acid is one of the bioactive metabolites from PG peel that shows the highest extraction efficiency using supercritical fluid [47, 56].

### 2.2. Polyphenolic Compounds

#### 2.2.1. Flavonoids

**Figure 2:**
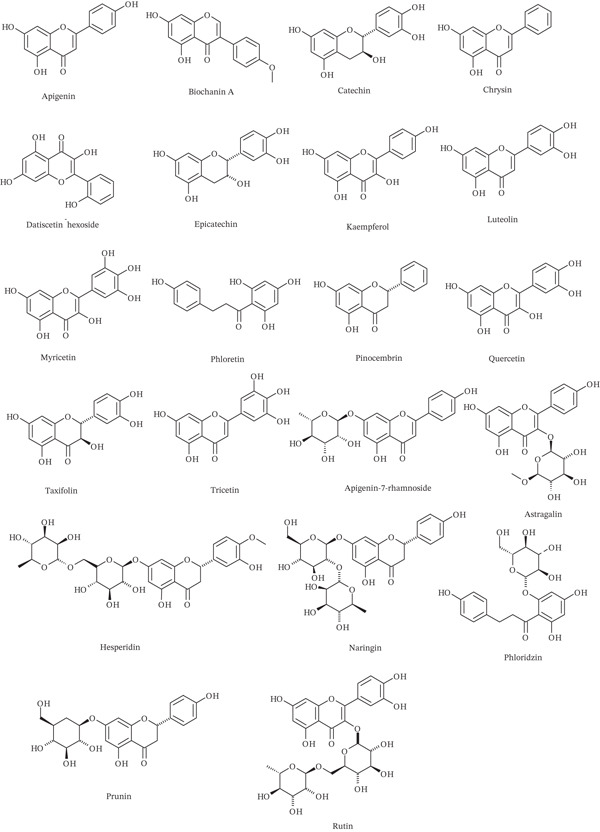
Chemical structures of flavonoid compounds isolated from *Punica granatum.*

Flavonoids are one of the most abundant groups of secondary metabolites in *P. granatum*. Structurally, they are composed of 15 carbon atoms organized into three rings: A and B, which are aromatic six‐membered rings, connected by C, a heterocyclic ring containing three carbon atoms and one oxygen atom. These compounds exhibit significant pharmacological activities, such as antimicrobial, antioxidant, antiviral, anti‐inflammatory, and anticancer properties. Among the flavonoids isolated from *P. granatum*, notable examples include tricetin, pinocembrin, quercetin, phloretin, naringin, myricetin, kaempferol, apigenin, biochanin A, apigenin‐7‐rhamnoside, astragalin, datiscetin–hexoside, hesperidin, rutin, phloridzin, taxifolin, epicatechin, chrysin, catechin, and prunin, among others (Figure 2). There are more than 55 flavonoids identified in the plant [6, 14, 15, 57–61].

#### 2.2.2. Anthocyanins and Proanthocyanidins

**Figure 3:**
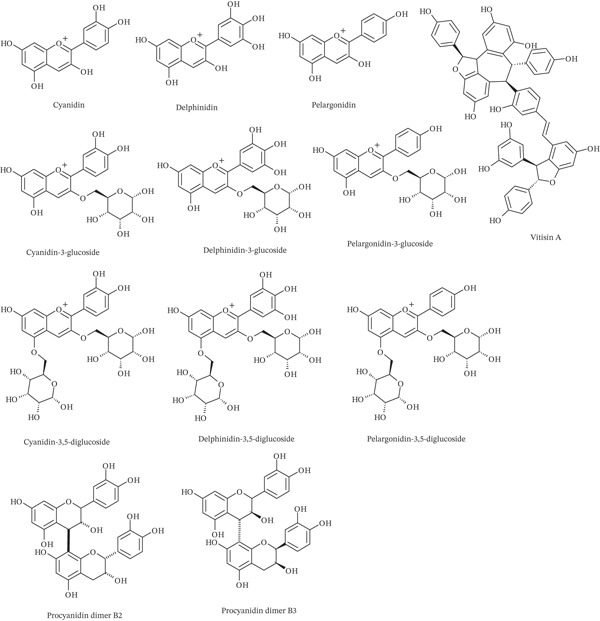
Chemical structures of anthocyanin and proanthocyanidin compounds isolated from *Punica granatum.*

Anthocyanins are colorful secondary metabolites of the flavonoid type with a basic structure of flavylium or flavylium cation, responsible for the red and orange colors in *P. granatum*. These compounds exhibit significant antioxidant properties, making them nutritionally and health‐relevant as they help combat oxidative stress in cells. Anthocyanins also have applications in the food industry as natural colorants. The anthocyanins identified in *P. granatum* include cyanidin 3‐glucoside, cyanidin 3,5‐diglucoside, delphinidin 3‐glucoside, delphinidin 3,5‐diglucoside, pelargonidin 3‐glucoside, pelargonidin 3,5‐diglucoside, and vitisin A [14, 62–66], whereas among the proanthocyanidins, procyanidin dimers B2 and B3 have been identified (Figure 3) [60].

#### 2.2.3. Phenolic Acids

**Figure 4:**
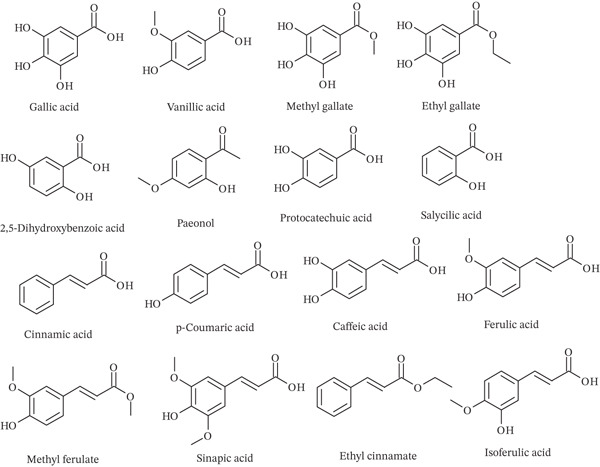
Chemical structures of phenolic acid compounds isolated from *Punica granatum.*

Phenolic acids are secondary metabolites that possess a carboxyl group attached to an aromatic ring. They are classified as derivatives of benzoic acid and cinnamic acid. These compounds are particularly interesting as bioactive molecules due to their high antioxidant capacity, which stems from their ability to donate electrons and neutralize free radicals. They exhibit functions such as antimicrobial, anti‐inflammatory, antitumor, and cardioprotective activities. Among the phenolic acid derivatives present in *P. granatum* are gallic acid, vanillic acid, methyl gallate, ethyl gallate, protocatechuic acid, salicylic acid, cinnamic acid, coumaric acid, caffeic acid, ferulic acid, sinapic acid, and others (Figure 4) [6, 42, 60, 67, 68].

#### 2.2.4. Tannins

**Figure 5:**
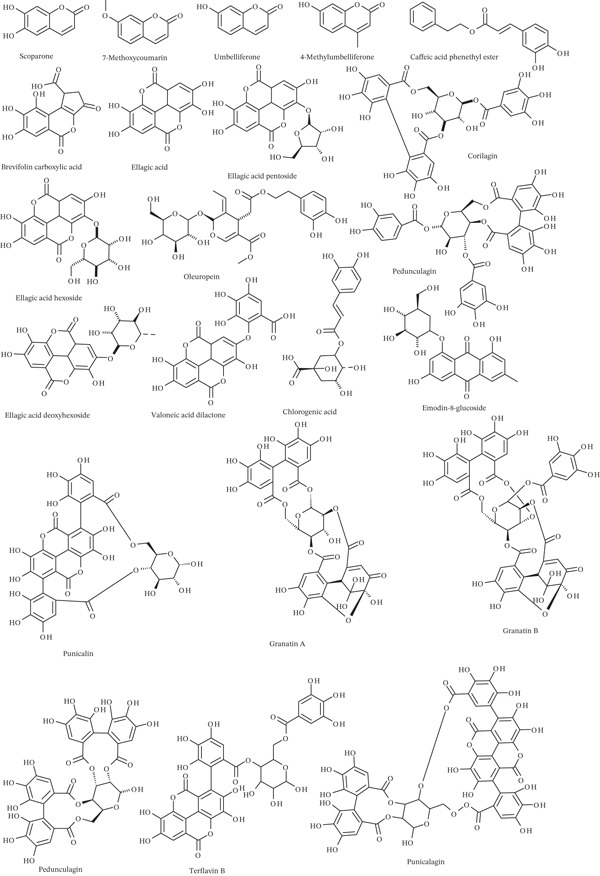
Chemical structures of tannin compounds isolated from *Punica granatum* (Part 1).

**Figure 6:**
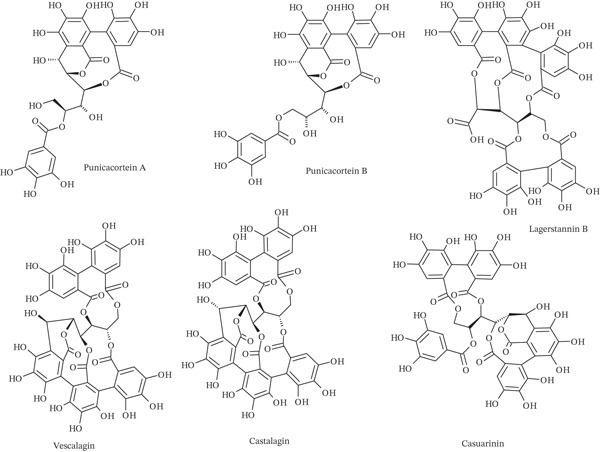
Chemical structures of tannin compounds isolated from *Punica granatum* (Part 2).

Tannins are phenolic compounds derived from secondary metabolism, primarily formed by the bonding of simple phenolic acids such as gallic acid and ellagic acid. Like other phenolic acids, these compounds exhibit significant antioxidant, antimicrobial, and anti‐inflammatory activities. This group of metabolites is especially important in *P. granatum* (PG), as they are found throughout the plant, from its seeds to its peels. Among the most commonly identified tannins in *P. granatum* are ellagic acid and its derivatives, valoneic acid dilactone, pyrogallol, oleuropein, chlorogenic acid, corilagin, pedunculagin, granatin A and B, punicalin *α* and *β*, casuarinin, castalagin, scoparone, brevifolin carboxylic acid, umbelliferone, punicatannin, and punicafolin, among others (Figures 5 and 6) [6, 14, 69–71].

#### 2.2.5. Lignans

**Figure 7:**
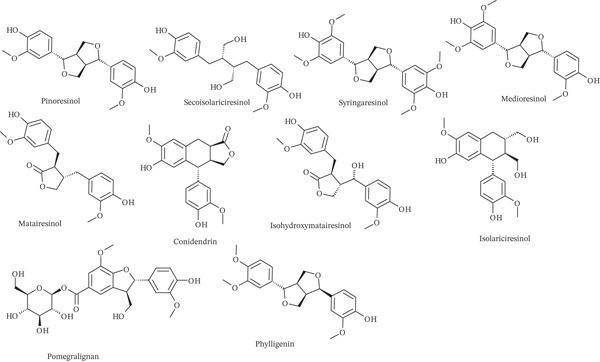
Chemical structures of lignan compounds isolated from *Punica granatum.*

Lignans are polyphenolic compounds derived from secondary metabolism, formed by the linkage of two phenylpropanoid units primarily connected through an 8–8 ^′^ bond. Lignans offer health benefits such as cardioprotective, antioxidant, antitumor, and anti‐inflammatory properties. *P. granatum* (PG) contains several lignans, including pinoresinol, secoisolariciresinol, syringaresinol, cyclolariciresinol, matairesinol, medioresinol, conidendrin, iso‐hydroxymatairesinol, isolariciresinol, phylligenin, and pomegralignan (Figure 7) [43, 72, 73].

### 2.3. Alkaloids

**Figure 8:**
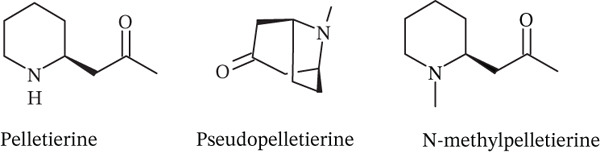
Chemical structures of alkaloid compounds isolated from *Punica granatum.*

Alkaloids are compounds derived from secondary metabolism that contain one or more nitrogen atoms, typically in a cyclic system. Due to their high solubility in water, they are usually easily absorbed and exhibit multiple pharmacological effects, such as analgesic, stimulant, and depressant of the central nervous system and antitumor, antispasmodic, antihypertensive, and antimicrobial activities. In the case of *P. granatum*, there are few alkaloids present, with pelletierine derivatives standing out, such as pseudopelletierine, N‐methylpelletierine, and di‐pelletierine (Figure 8) [15, 43, 64].

### 2.4. Terpenes

**Figure 9:**
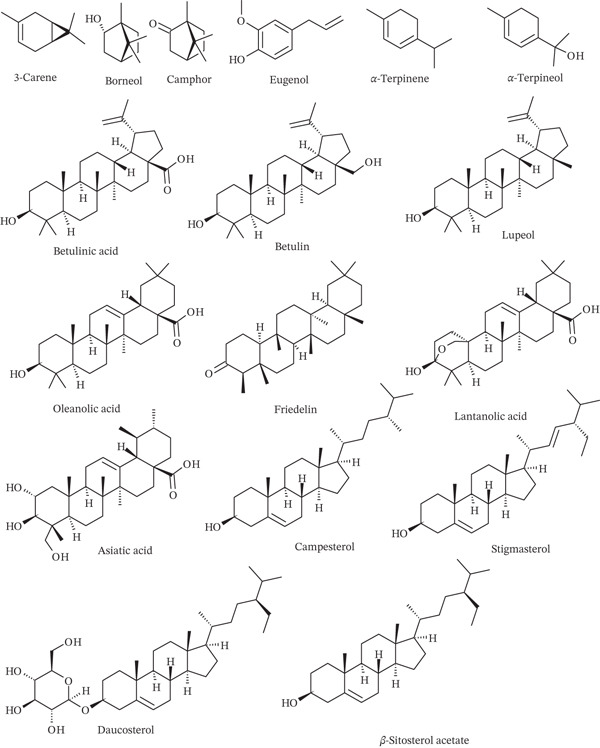
Chemical structures of terpene compounds isolated from *Punica granatum.*

Terpenes are the most diverse and abundant group among secondary metabolites. Structurally, they are composed of isoprene units linked together. This remarkable structural versatility allows them to have multiple therapeutic applications, including anti‐inflammatory, antioxidant, antimicrobial, anticancer, analgesic, and bronchodilator properties. In the case of *P. granatum*, the most important terpenes identified include eugenol, camphor, borneol, 3‐carene, *α*‐terpinene, *α*‐terpineol, betulin, betulinic acid, lupeol, oleanolic acid, friedelin, lantanolic acid, asiatic acid, campesterol, stigmasterol, daucosterol, and *β*‐sitosterol acetate (Figure 9) [14, 60, 74–76].

### 2.5. Fatty and Organic Acids

**Figure 10:**
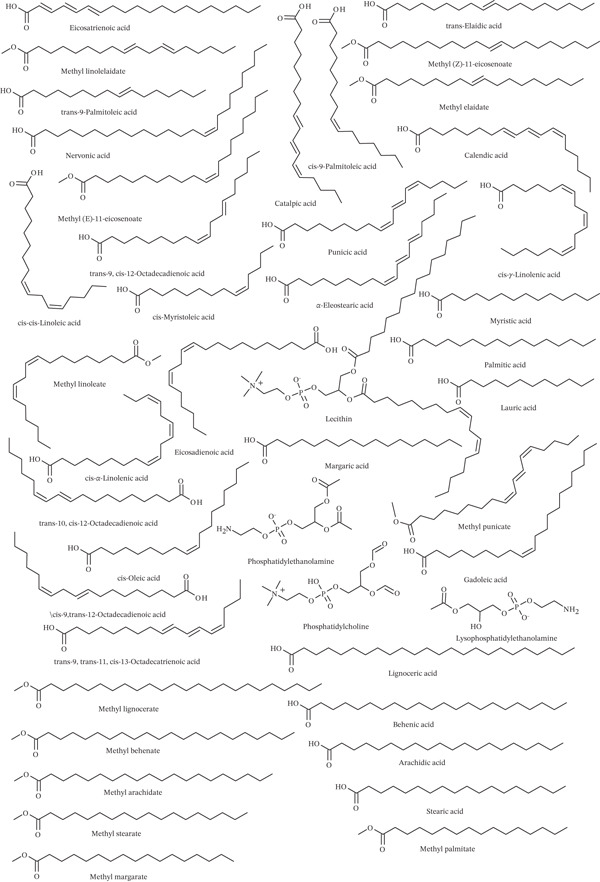
Chemical structures of fatty and organic acid compounds isolated from *Punica granatum.*

Fatty acids and organic acids are metabolites formed by hydrocarbon chains of variable length, with a carboxyl group at one end for fatty acids and one or more carboxyl groups at their ends for organic acids. These types of metabolites exhibit a wide variety of pharmacological effects, including antimicrobial, antioxidant, insecticidal, anthelmintic, and antinociceptive activity. In the case of *P. granatum*, a great variety of acids can be found, including punicic acid, *α*‐eleostearic acid, *cis*‐*α*‐linolenic acid, *cis*‐*γ*‐linolenic acid, calendic acid, *trans*‐9, *trans*‐11, *cis*‐13‐octadecatrienoic acid, catalpic acid, *cis*‐9‐palmitoleic acid, *cis*‐oleic acid, *trans*‐elaidic acid, *cis*‐*cis*‐linoleic acid, *trans*‐9, *cis*‐12‐octadecadienoic acid, *trans*‐10, *cis*‐12‐octadecadienoic acid, gadoleic acid, *cis*‐9,*trans*‐12‐octadecadienoic acid, *cis*‐myristoleic acid, *trans*‐9‐palmitoleic acid, eicosatrienoic acid, methyl (E)‐11‐eicosenoate, nervonic acid, methyl (Z)‐11‐eicosenoate, methyl linolelaidate, methyl elaidate, methyl linoleate, methyl punicate, lecithin, *trans*‐elaidic acid, eicosadienoic acid, phosphatidylethanolamine, phosphatidylcholine, lysophosphatidylethanolamine, lauric acid, myristic acid, palmitic acid, margaric acid, stearic acid, arachidic acid, behenic acid, lignoceric acid, methyl palmitate, methyl margarate, methyl stearate, methyl arachidate, methyl behenate, and methyl lignocerate (Figure 10) [77–86].

### 2.6. Structural Analysis of Phytochemicals Associated With Antiparasitic Activity

Beyond the qualitative identification of metabolites, the phytochemical profile of *P. granatum* suggests several structure‐related features associated with antiparasitic activity. Hydrolyzable tannins and ellagitannins, particularly punicalagin and punicalin, possess multiple hydroxyl groups that confer strong protein‐binding capacity, metal‐chelating properties, and the ability to disrupt membrane‐associated proteins [87, 88], which may explain their activity against protozoa and helminth teguments. Phenolic acids such as gallic and ellagic acids exhibit redox‐modulating properties that can promote oxidative imbalance in parasites while also contributing to host antioxidant protection [32, 89]. Flavonoids, including quercetin, catechin, luteolin, and naringenin, contain aromatic hydroxylated scaffolds capable of interacting with enzymes involved in energy metabolism, nucleic acid synthesis, and cytoskeletal organization [90–94]. In addition, amphiphilic constituents such as saponins and sterols may alter membrane permeability through interactions with lipid bilayers [95, 96], whereas alkaloids and quinones may contribute through neuromuscular interference or redox cycling [97, 98]. Collectively, these complementary structural properties may underlie the broad antiparasitic spectrum of PG extracts and their reported additive or synergistic effects with conventional drugs, as discussed in Sections 3 and 4.

## 3. Effects of PG on Protozoa

**Figure 11:**
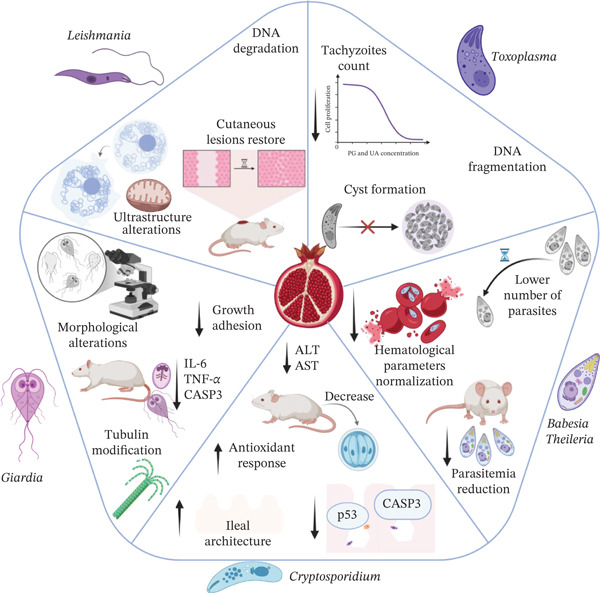
Schematic representation of the effects of pomegranate compounds against pathogenic protozoa. In vitro studies showed that polyphenols induce growth impairment and interact with key parasite targets, including the cytoskeleton, DNA, and differentiation processes. In animal models, pomegranate administration reduces parasitemia, modulates the immune response, or enhances host recovery by improving the antioxidant profile/restoring tissue architecture. Created in https://BioRender.com.

Pathogenic protozoa are significant etiological agents responsible for heterogeneous diseases affecting millions worldwide. Notable examples include *Leishmania* spp., *Toxoplasma* spp., *Giardia lamblia*, and *Cryptosporidium* spp. [99]. These infections lead to substantial morbidity and mortality, particularly in low‐ and middle‐income countries [100]. Beyond health impacts, these diseases impose considerable economic burdens due to healthcare costs and lost productivity [101]. Despite their prevalence, many protozoan infections are often neglected, receiving less attention and funding compared to other infectious diseases. This neglect hampers the development of effective diagnostics, treatments, and control measures, perpetuating cycles of infection and poverty in affected regions [102]. Addressing pathogenic protozoan diseases requires a concerted global effort to enhance research, improve healthcare infrastructure, and implement sustainable prevention and control strategies [103]. In the following subthemes, the significant antiparasitic activity against pathogenic protozoa of PG compounds is described, both in vitro and in vivo. In vitro studies have shown that these compounds impair parasite growth and interact with molecular targets, which are essential for parasite survival and proliferation. Additionally, in vivo models have highlighted the potential therapeutic benefits of phytochemicals, which reduce parasitemia, modulate the immune response, and enhance host recovery. These effects are mediated through improvements in the antioxidant profile and the restoration of tissue architecture, suggesting that polyphenols may serve as promising candidates for the development of novel antiparasitic strategies (Figure 11).

### 3.1. Antileishmanial Effect

Leishmaniasis is a neglected tropical disease, with *Leishmania infantum* being the etiological agent of the cutaneous (CL) and visceral (VL) forms of this zoonotic disease. Leishmaniasis affects over 1 billion people in endemic regions and results in an estimated 30,000–1 million new cases annually [104]. One of the primary challenges in combating leishmaniasis is the complexity of its transmission dynamics, involving multiple *Leishmania* species, diverse sandfly vectors, and various animal reservoir hosts. This complexity complicates the development of universal diagnostic tools, treatments, and preventive measures [105]. Moreover, the disease is often overlooked in public health agendas, leading to insufficient funding and research dedicated to its control [106].

The activity of PG peel extract against *L. infantum* promastigotes was evidenced by their antiproliferative effect with an IC_50_ (72 h) of 52.4 *μ*g/mL. The most relevant findings were DNA degradation and ultrastructure alterations in the protozoa, particularly in mitochondria (swelling) and nucleus (membrane rupture and chromatin changes). Interesting vacuoles without content were also found in the presence of the extract. In this study, the main compounds identified in the extract were hydrolyzable tannins, particularly *α* and *β*‐punicalagin [107]. The fruit of PG was also effective on cutaneous leishmaniasis in vitro and in vivo. Using PG juice, a growth inhibition in *Leishmania major* promastigotes was determined (IC_50_ of 118.2 *μ*g/mL). In female BALB/c mice infected with the protozoa, the administration of PG as preventive (before infection) caused the restoration of cutaneous lesions of infected mice. The use of PG as treatment alone or in combination with ciprofloxacin resulted in an improvement in injuries, liver functions, and oxidant/antioxidant imbalance. Authors suggest that these activities are due to the presence of luteolin, ellagitannins, and epigallocatechin gallate [27].

### 3.2. Antitoxoplasma Effect

Toxoplasmosis is a neglected infection whose most severe effects include serious brain complications and death. The most vulnerable groups are immunocompromised individuals, children, and pregnant women [108]. This parasitosis affects warm‐blooded animals, and regarding its impact on humans, it is estimated that it affects a third of the population worldwide [109]. A study demonstrated the use of a PG extract and the supplementation with urolithin‐A (UA) (a metabolite produced after consumption of PG) as neurotoxoplasmosis agents. Results of in vitro assays evidenced that treatments with the extract and UA caused a reduction in the number of parasites (tachyzoites) in addition to affecting cyst formation in differentiated human neuronal cells. In a murine chronic toxoplasmosis model, the administration of UA confirmed the interference in cyst formation. The authors relate this effect to an alteration in the behavioral manipulation. In infected animals, an unusual attraction to cat odor is observed. Interestingly, the treatment with UA reduces this behavior [110]. Another study reported the effects of an aqueous extract of *P. granatum* (PG) peel against *Toxoplasma gondii* tachyzoites in vitro, demonstrating growth inhibition with an IC_50_ of 400–500 *μ*g/mL. The extract also exhibited low toxicity toward host cells (HFF), with a selectivity index (SI) of 2.4. Phytochemical analysis revealed that the extract contains high levels of phenols, gallic tannins, and sterols. Furthermore, the extract induced apoptosis‐like effects in *T. gondii* tachyzoites, evidenced by phosphatidylserine exposure on the tachyzoite surface and DNA fragmentation [111].

### 3.3. Antibabesial and Antitheilerial Activities

Theileriosis and babesiosis are significant tick‐borne diseases affecting livestock and, in some cases, humans, leading to substantial economic losses and health concerns worldwide [112]. The complex life cycle of *Theileria*, involving both mammalian hosts and tick vectors, complicates control measures. Challenges in managing theileriosis include the emergence of drug‐resistant strains and the need for effective vaccines to curb its spread [113]. Babesiosis, caused by *Babesia* species, affects a range of hosts, including domestic, wildlife, and human hosts. Control efforts are hindered by the persistent carrier state in recovered animals and the lack of highly effective vaccines [114, 115].

The effect of a methanolic extract of PG peel was demonstrated. In vitro, PG significantly reduced the number of *Theileria equi* parasites, with an IC_50_ of 100 ± 16.20 * μ*g/mL. Additionally, it exhibited inhibitory activity against several *Babesia* species, with IC_50_ values of 154.45 ± 23.11 * μ*g/mL for *Babesia bovis*, 40.90 ± 9.35 * μ*g/mL for *Babesia bigemina*, 72.71 ± 14.77 * μ*g/mL for *Babesia divergens*, and 77.27 ± 16.94 * μ*g/mL for *Babesia caballi*. The combination of PG with diminazene aceturate (an antibabesial drug) on *B. bovis* revealed a synergistic or additive interaction, using high or low concentrations of the extract, respectively. The in vivo effect of PG was determined against mice infected with *Babesia microti*. Using a combination therapy (with lower doses of PG) showed a considerable reduction in parasitemia and treated hemolytic anemia associated with babesiosis [116].

The antipiroplasm activity of the methanolic peel extract may be associated, at least in part, with polyphenolic constituents commonly reported in PG peel, including ellagitannins, flavonoids, and phenolic acids. These metabolites have been linked to membrane disruption, oxidative stress induction, and interference with metabolic pathways [117–120], essential for intraerythrocytic parasite replication. The synergistic or additive interaction observed with diminazene aceturate further suggests complementary mechanisms of action that may enhance antiparasitic efficacy.

### 3.4. Anticryptosporidial Effect

Cryptosporidiosis, caused by the protozoan parasites *Cryptosporidium parvum* and *Cryptosporidium hominis*, is a significant global health concern, particularly affecting children under 2 years of age in low‐ and middle‐income countries [121]. Transmission occurs primarily through the fecal–oral and the resilience of *Cryptosporidium* oocysts against common disinfectants, including chlorine, poses challenges for water treatment and infection control. In addition, the current pharmacological treatments are not safe for infants [122].

The effects of white and red peel extracts of *P. granatum* (PG), prepared from ultrafine plant powder, were evaluated in a murine model infected with *C. parvum*. Both extracts reduced oocyst shedding and minimized histopathological alterations in the ileum. Notably, the red peel extract was more effective in restoring normal ileal architecture. Biochemically, the red PG peel extract improved serum albumin and globulin levels in infected animals. It also reduced elevated ALT and AST levels associated with parasite infection. These improvements were observed 1 week post‐treatment. Additionally, both extracts enhanced the antioxidant response in infected mice, with significant effects noted after 2 weeks of treatment. The effect could be attributable to gallic acid, chlorogenic acid, catechin, quercetin, caffeine, caffeic acid, syringic acid, and naringenin [123].

Other authors recently probed the effect of a methanolic PG peel extract—rich in phenols and saponins—on a cryptosporidiosis mouse model. In infected animals treated with PG, a decrease in intestinal epithelial lining degeneration and in the presence of oocysts within gastric glands was found. In addition, the expression of p53 and caspase‐3 in the glandular gastric mucosa was diminished in comparison with the infected group. They also demonstrated that treatment with PG caused a protective effect against splenic damage induced by the infection [124].

### 3.5. Antigiardiasic Effect

*G. lamblia* is an intestinal protozoan that infects humans, companion animals, livestock, and wildlife and causes diarrhea, malabsorption, weight loss, and other complications [125]. Controlling giardiasis presents several challenges, including the emergence of drug‐resistant strains, suboptimal dosing regimens, and limited efficacy of current treatments in certain populations [126]. Moreover, the resilience of *Giardia* cysts against common disinfectants, such as chlorine, complicates water treatment efforts, necessitating alternative methods for effective inactivation [127]. Urgent efforts are needed to develop novel therapeutics to control giardiasis and reduce its global burden.

The hydroalcoholic PG peel extract demonstrated a significant antigiardial effect in vitro, with an IC_50_ of 179 *μ*g/mL, inhibiting trophozoite growth and adhesion. The most notable effects included morphological alterations—such as elongation, shape disturbances, and caudal and flagellar deformations—and changes in the expression and distribution of tubulin, suggesting an interaction with the *Giardia* cytoskeleton. The extract′s composition included ellagic acid, punicalin, punicalagin, and luteolin [41]. The effect of PG on mice infected with *G. lamblia* revealed its potential as a preventive and therapeutic agent. The administration of methanolic extract lowered the shedding of cysts at Day 20, in mice treated before and after the infection, with reductions of 87.2% and 75.6%, respectively. A reduction in the presence of *Giardia* antigen was also evidenced [128]. In another study, the activity of PG ethanolic extract was evaluated on rats infected with *Giardia*. As a result, PG reduced the number of parasites (cysts and trophozoites), diminished the expression of proinflammatory cytokines (IL‐6 and TNF‐*α*), and increased NO production. Furthermore, the treatment with PG protected the intestine of infected animals, due to lower expression of caspase‐3 and a marked recovery in the intestinal mucosa, in comparison with untreated infected rats [129].

## 4. Effects of PG on Helminths

**Figure 12:**
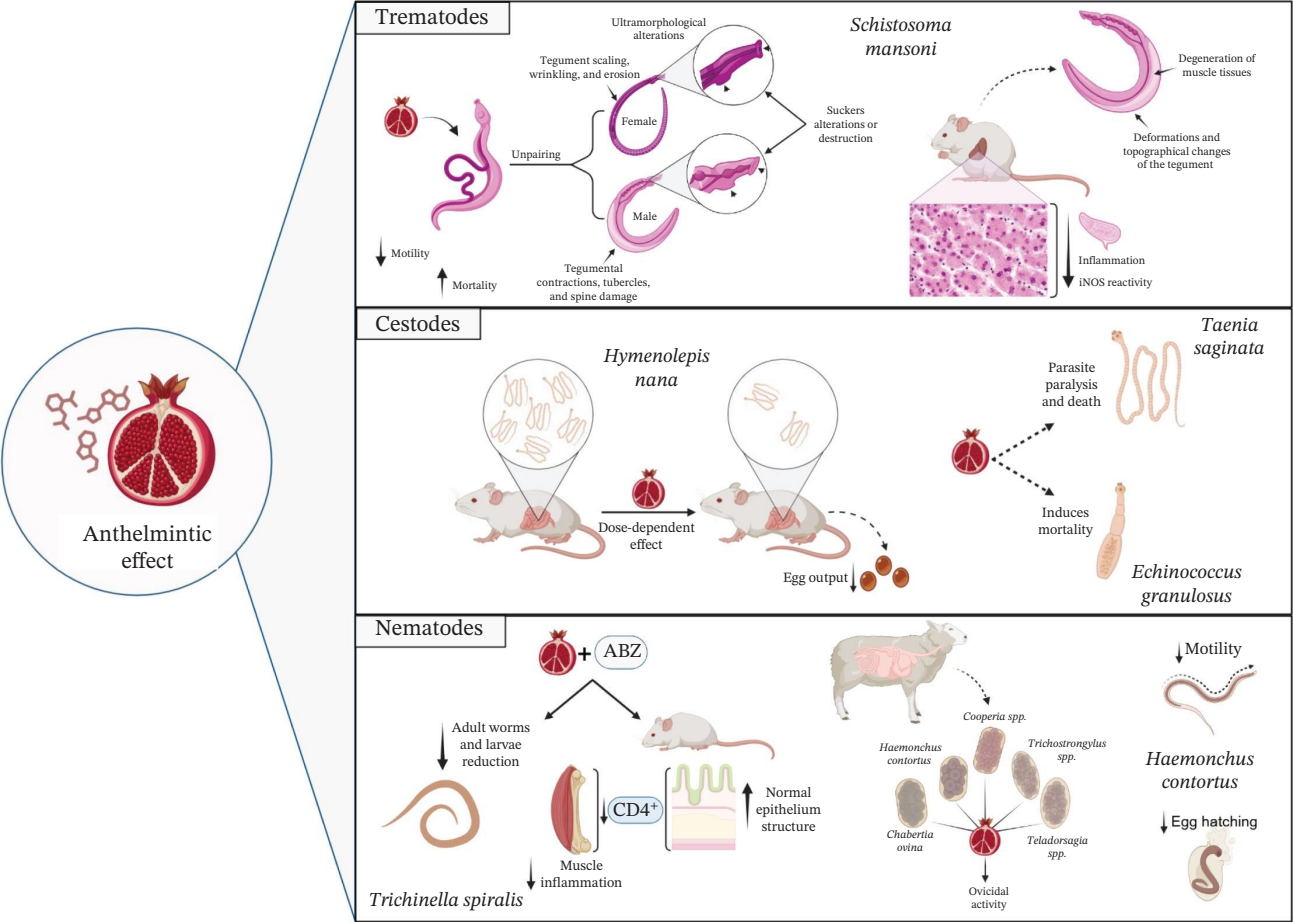
Anthelmintic effects of *Punica granatum* extracts on trematodes, cestodes, and nematodes. Extracts impair parasite motility, induce morphological alterations, reduce egg viability, and enhance host immune responses, demonstrating potential as a complementary or alternative anthelmintic agent. Created in https://BioRender.com.

Parasitic helminths, classified into trematodes, cestodes, and nematodes, cause significant morbidity and socioeconomic burdens worldwide, which compromise human and other animal health [130, 131]. Helminth infections predominantly affect marginalized, low‐income populations in resource‐limited regions. The resulting morbidity creates a cycle of illness, poverty, reduced productivity, and hindered socioeconomic progress [132]. This section describes the effects of PG on various helminths. Studies have shown that PG extracts cause structural damage, impair motility, and reduce parasite viability, often in a concentration‐ and time‐dependent manner (Figure 12 and Table 1).

**Table 1:** Antiparasitic effects of *Punica granatum* L. bioactive compounds: Summary of their principal effects from in vitro and in vivo studies.

Used product (plant part and extraction method)	Identified compounds	Affected pathogen	Key findings	References
Aqueous peel extract obtained by maceration and heat	*α*‐ and *β*‐punicalagin, *α*‐ and *β*‐punicalin, ellagic acid, and ellagic acid derivatives	*Leishmania infantum*	Affect promastigotes with an IC_50_ (72 h) of 52.4 *μ*g/mL. DNA degradation and ultrastructure alterations	Imperatori et al. [107]
Juice of fruit, filtered and diluted in water (1:1 *v*/*v*)	Nonidentified	*Leishmania major*	Inhibited promastigotes (IC_50_ 118.2 *μ*g/mL) in vitro. Restored lesions as a preventive agent and improved histopathological conditions, liver function, and antioxidant activity as a therapeutic agent, in a murine infection model	Alkathiri et al. [27]
Pomella PE (Verdure Sciences)	Urolithin‐A (Tocris Bioscience)	*Toxoplasma gondii*	Inhibition of tachyzoites and impaired cyst formation in vitro. In a chronic toxoplasmosis murine model, UA also affects cyst formation and reduces the behavioral manipulation induced by the parasite	Tan et al. [110]
Aqueous extract of peel (relation 1:10 *m*/*v*) by heating and stirring	The main families were phenols, gallic tannins, and sterols. Also identified coumarins, flavonoids, and saponins	*Toxoplasma gondii*	Diminished tachyzoites in vitro (IC_50_ 400–500 *μ*g/mL) without affecting host cells (SI of 2.4). Also causes apoptosis‐like cell death, evidenced by phosphatidylserine externalization and DNA fragmentation	Elkoraichi et al. [111]
Methanolic peel extract (1:5 *m*/*v* relation)	Nonidentified	*Babesia bovis*, *B. bigemina*, *B. divergens*, *B. caballi*, and *Theileria equi* in vitro. *B. microti* in vivo	The extract inhibits growth alone or combined with diminazene aceturate, showing synergistic or additive effects depending on concentration in vitro. Infected mice with *B. microti* receiving combination therapy exhibited reduced parasitemia and improved parasite‐related hemolytic anemia	Eltaysh et al. [116]
Aqueous extracts of white and red peel (1:10 *m*/*v* relation) using microwave	Gallic acid, chlorogenic acid, catechin, quercetin, caffeine, caffeic acid, syringic acid, and naringenin	*Cryptosporidium parvum*	Reduction in oocyst shedding was observed in a murine infection model, with a greater effect seen in the red peel extract. *Punica granatum* improved histopathological alterations, as well as biochemical and antioxidant parameters	Aboelsoued et al. [123]
Methanolic extract of the peel (2:5 *m*/*v* relation) by mashing	Phenols, saponins, flavonoids, glycosides, tannins, reducing sugars, and alkaloids	*Cryptosporidium parvum*	The extract demonstrated protective effects against gastric and splenic damage caused by the cryptosporidiosis mouse model. It also reduced the expression of p53 and caspase‐3 in the glandular gastric mucosa	El‐Shewehy et al. [124]
Hydroalcoholic (30% ethanol) peel extract (1:12 *m*/*v* relation) by microwave/ultrasound hybrid system	Ellagitannins (punicalin and punicalagin isomers and galloyl‐dihexahydroxydiphenoyl‐hexoside), flavones (luteolin 6‐C‐glucoside), and ellagic acid (including ellagic acid hexoside)	*Giardia lamblia*	The extract reduced the growth (IC_50_ 179 *μ*g/mL) and adhesion of trophozoites in vitro, affecting microtubule‐rich structures and causing morphological disturbances in the parasite. Additionally, it altered the expression and distribution of *α*‐tubulin	Palomo‐Ligas et al. [41]
Methanolic (70%) peel extract obtained by percolation	Nonidentified	*Giardia lamblia*	The extract exerted both preventive and therapeutic effects in a murine model of infection, reducing cyst count and parasite antigen detection	Al‐Megrin [128]
Ethanolic peel extract (relation 1:5 *m*/*v*) obtained by maceration	Nonidentified	*Giardia lamblia*	The extract reduced the viability of human cysts in vitro. In a rat model of giardiasis, it also decreased the number of cysts and trophozoites, reduced inflammatory cytokines, and improved intestinal architecture	El‐Kady et al. [129]
Ethanolic extracts from leaves and stem bark obtained by maceration	Polyphenols, glycosides, triterpenes, sterols, flavonoids, anthocyanins, triglycerides, tannins, and alkaloids	*Schistosoma mansoni*	The extracts disrupted *S. mansoni* mating, reduced motility, and induced mortality in vitro, with pronounced tegumental damage. In vivo, they reduced liver inflammation, eliminated eggs, and modulated iNOS expression	Yones et al. [133]
Ellagitannins were separated from aqueous extracts of rind, placenta, stem bark, and root bark using ion chromatography. The compounds were eluted with methanol	Ellagitannins	*Schistosoma mansoni*	Ellagitannins from *Punica granatum* rind caused 100% mortality of worms, inducing morphological and histopathological damage, including tegumental disruption, sucker impairment, and motor dysfunction	Abozeid et al. [18]
Methanolic extract of peel obtained by percolation	Nonidentified	*Hymenolepis nana*	PG peel extract reduced egg output in feces and worm burden in the intestines of *H. nana*‐infected mice	Al‐Megrin [38]
Decoction of peel	Alkaloids, flavonoids, tannins, saponins, and quinones	*Taenia saginata*	The decoctions caused paralysis and death of the parasite in a concentration‐dependent manner	Saptarini and Mustarichie [134]
Extract from blossoms (relation 1:5 *m*/*v*) using ethyl liquor	Nonidentified	*Echinococcus granulosus*	The extract showed concentration‐dependent lethality against *Echinococcus granulosus* protoscolices (LC_50_: 6.6–11.1 mg/mL)	Hussein and Al‐Almubark [135]
Ethanolic extract from the peel obtained by maceration	Nonidentified	*Trichinella spiralis*	The extract enhances the therapeutic efficacy of the reference drug and exhibits immunomodulatory effects, as well as ultrastructural improvements in the intestines of parasitized animals	Esmat et al. [136]
Aqueous extract of the fruit and rind by maceration	Tartaric acid, mannitol, glucuronic acid, 2,3‐(S)‐hexahydroxyphenyl‐D‐glucose, gallic acid, phelligridin J, valoneic acid dilattone, syringic acid, ellagic acid, and ducheside A	Gastrointestinal nematodes in sheep (*Trichostrongylus* spp., *Haemonchus contortus*, *Teladorsagia* spp., *Chabertia ovina*, and *Cooperia* spp.)	Pomegranate extracts and fractions showed significant ovicidal activity against nematode eggs	Castagna et al. [137]
Methanolic peel extract using cold maceration	Alkaloids, tannins, flavonoids, glycosides, and phenols	*Haemonchus contortus*	Crude extract causes mortality and egg hatching inhibition in the nematode	Ahmed et al. [138]

### 4.1. Antitrematodal Activity

Trematodes, a diverse group within the phylum Platyhelminthes, are parasitic flatworms that infect a wide range of vertebrate hosts, including humans. These parasites are responsible for significant global health challenges, particularly in developing countries [139]. Schistosomiasis is a parasitic disease caused by fluke flatworms of the genus *Schistosoma*. According to the World Health Organization, it is the second most prevalent parasitic disease, after malaria [140]. The activity of four extracts from the leaves and stem bark of both edible *P. granatum* L. and ornamental *P. granatum* L. var. *nana* was evaluated in vitro and in vivo. In *Schistosoma mansoni* adult worms, the extracts disrupted the natural mating process by causing the separation of paired schistosomes. Additionally, the extracts reduced motility and increased worm mortality. The edible PG extract induced more pronounced ultrastructural changes, particularly in the tegument of male worms, and caused scaling, erosion of the dorsal region, and alterations in the oral and ventral suckers of female worms. In vivo, the extracts reduced liver inflammation and led to the absence of eggs at the highest dose. The expression of iNOS was also modulated by the extracts, with the stem bark of edible PG showing negative reactivity. Phytochemical analysis revealed the presence of polyphenol glycosides, triterpenes, sterols, flavonoids, anthocyanins, triglycerides, tannins, and alkaloids in both extracts [133].

In another study, the antiparasitic effect of *P. granatum* was evaluated using ellagitannins extracted from the fruit rind, placenta, stem bark, and root bark. These compounds were tested on *S. mansoni* adult worms recovered from mice infected with the trematode. Ellagitannins from the rind exhibited the highest activity, achieving 100% parasite mortality. Morphological alterations in treated worms were observed, including head deformations, surface corrugations, and structural damage. Histopathological analysis revealed significant muscle tissue damage accompanied by vacuolization in worms exposed to rind‐derived ellagitannins. Additionally, the ellagitannins caused severe tegumental damage, impaired the function of suckers, and disrupted motor activity [18].

### 4.2. Cestocidal Activity

Cestode infections, such as diphyllobothriosis and taeniosis, are globally distributed, affecting both developing and developed nations. The specific causative species vary considerably across different geographical regions. Additionally, increased globalization has contributed to the complexity of their epidemiology, influencing transmission dynamics and distribution patterns [141].

*Hymenolepis nana* is a parasitic tapeworm with a worldwide distribution, and it mainly affects countries with inadequate sanitary conditions [142]. The effects of PG peel extract were evaluated in mice infected with *H. nana*. The authors reported that administration of the extract reduced egg output in the fecal pellets of infected animals in a dose‐dependent manner. Additionally, necropsy findings revealed a reduction in the number of worms in the intestines of treated mice [38].

*Taenia solium*, *Taenia saginata*, and *Taenia asiatica* are taeniid tapeworm species responsible for causing taeniasis in humans and cysticercosis in their intermediate host animals [143]. The economic impact of taeniasis and its complications is substantial, affecting both human health and the livestock industry [144]. The activity of PG peel extract was also demonstrated against *T. saginata*. Different concentrations of decoctions used for obtaining the peel extracts exhibited varying levels of efficacy. At the highest concentrations (75% and 100%), the extract caused parasite death. Additionally, helminth paralysis was observed, with this effect increasing in a concentration‐dependent manner. Phytochemical analysis identified several secondary metabolites in PG, including alkaloids, flavonoids, tannins, saponins, and quinones, which are believed to contribute to its anthelmintic activity [134].

In another study, the extract of PG flowers demonstrated antiparasitic activity against the protoscolices of *Echinococcus granulosus*. The extract induced mortality in a concentration‐dependent manner. The LC_50_ (lethal concentration required to kill 50% of the protoscolices) was 11.1 mg/mL after 15 min of exposure and decreased to 6.6 mg/mL after 60 min. Similarly, the median lethal time (LT_50_) was 79.4 min at the lowest concentration tested, decreasing to 18.3 min at the highest concentration. Although the phytochemical composition was not identified in this study, the authors noted that PG is known to contain phenolic compounds and anthocyanins [135].

### 4.3. Nematicidal Activity

Nematode infections, particularly those caused by soil‐transmitted helminths (STHs) and hookworms, pose a significant global health burden [145]. These infections are especially prevalent among school‐aged children, leading to malnutrition, anemia, and impaired cognitive development [146].

Trichinellosis is a foodborne zoonotic disease caused by *Trichinella* spp., including *Trichinella spiralis*. This parasitic disease is among the seven most infectious diseases in the world, and efforts are needed to improve treatment options [147]. PG peel extract has improved the therapeutic efficacy of albendazole (ABZ) in a murine model of trichinellosis. When infected animals were administered with PG and a half dose of ABZ, a reduction in the number of adult worms and larvae stage was detected. The combined treatment also improved the intestinal epithelium with normal villi and diminished inflammation in the infected intestine and muscle. This was also accompanied by a reduction in the expression of the CD4^+^ marker in both tissues [136].

To investigate the traditional use of PG against gastrointestinal nematodes in sheep, aqueous extracts were prepared from the fruit and rind. Additionally, three fractions were obtained based on their solubility in methanol. These treatments were tested in vitro on eggs collected from sheep naturally infected with gastrointestinal nematodes. The identified genera included *Trichostrongylus* spp., *Haemonchus contortus*, *Teladorsagia* spp., *Chabertia ovina*, and *Cooperia* spp. The study demonstrated that all fractions exhibited significant ovicidal activity, comparable to that of thiabendazole, a conventional anthelmintic drug. The activity was attributed to the presence of various phytochemicals, particularly tannins and phenolic compounds [137]. In another study, the in vitro effects of PG peel and root extracts were analyzed in *H. contortus*. The treatments caused a reduction in the motility of adult helminths and a decrease in egg hatching rates. The nematocidal effects of PG were concentration‐ and time‐dependent, with efficacy comparable to that of ABZ [138].

## 5. Translational Considerations and Limitations

### 5.1. Considerations on PAINS (Pan‐Assay Interference Compounds) in the Phenolic Compounds of *P. granatum*

In drug discovery and natural product research, it is essential to consider the phenomenon of PAINS. This term, coined by Baell et al., describes compounds that tend to generate false positives in biological activity screening assays due to nonspecific interference mechanisms, rather than genuine interaction with the molecular target of interest [148, 149]. These compounds can promiscuously interact with proteins, form colloidal aggregates, autofluoresce, chelate metal ions, or react covalently with assay components, generating misleading signals [150, 151].

The mechanisms by which these compounds can generate false positives include the following:

a. *Nonspecific chemical reactivity*: The orthodiphenol (catechol) groups present in ellagitannins and flavonoids can easily oxidize under assay conditions, generating electrophilic quinones that covalently react with thiol groups of proteins, including enzyme targets [152, 153]
b. *Formation of colloidal aggregates*: Hydrolyzable tannins, such as punicalagin and punicalin, tend to form supramolecular aggregates that can nonspecifically sequester proteins, inhibiting their activity without a genuine pharmacological interaction [154–156]
c. *Interference with detection systems*: Many polyphenols absorb in the UV‐Vis range or are fluorescent, interfering with absorbance‐based assays (e.g., MTT and NADH assays) or fluorescence‐based assays. Anthocyanins, responsible for the red color of PG, are particularly problematic in this regard [151, 157]
d. *Metal ion chelation*: The arrangement of hydroxyl groups allows chelation of metals such as iron or copper, which can interfere in metal‐dependent assays or generate apparent antioxidant activity that does not reflect a direct biological effect [158, 159]
e. *Redox interference*: Polyphenols can generate reactive oxygen species or act as antioxidants in cellular assays, altering the redox state and generating nonspecific signals in viability or inflammation assays [160, 161]

Some of the phenolic compounds present in *P. granatum* have structural characteristics that place them within the categories of compounds with potential interference. However, it should be noted that although polyphenols are potential PAINS, their possible therapeutic use should not be ruled out.

The fact that a compound behaves as a PAINS in a simplified biochemical assay does not automatically invalidate its therapeutic potential. Several lines of evidence support this caution: First, PAINS artifacts are largely assay‐dependent; in more complex biological systems (cell‐based assays, tissue models, or in vivo settings), nonspecific interference is often mitigated by serum proteins, membrane barriers, or cellular metabolism [152, 162]. Second, numerous approved drugs or clinical candidates (e.g., doxycycline, quercetin, and epigallocatechin gallate) show PAINS properties in vitro yet exhibit genuine pharmacological effects in living organisms [150, 163]. Third, polyphenols from *P. granatum* can act through mechanisms that bypass classical enzyme inhibition, such as modulation of transcription factors (NF‐*κ*B and Nrf2), gut microbiota conversion into bioactive metabolites (e.g., urolithins), or synergistic interactions within the whole extract [164, 165]. Finally, the application of orthogonal validation methods (detergent controls, reducing agents, metal chelators, and nonoptical detection techniques) allows researchers to discriminate between artifactual interference and true biological activity [163, 166]. Therefore, while PAINS flags should prompt rigorous experimental scrutiny, they should not be used as an outright exclusion criterion for natural products derived from *P. granatum*.

### 5.2. Synergistic Interactions and Gastrointestinal Bioavailability of PG Phytochemicals

In general, the biological activity of plant extracts is frequently attributed to the ability of their metabolites to modulate multiple molecular targets, including proteins, biomembranes, and nucleic acids, leading to altered membrane permeability, oxidative imbalance, neurotoxic effects, or disruption of essential cellular pathways [167]. This multitarget nature may represent an advantage in the context of parasitic diseases, as it could reduce the probability of resistance development. Consequently, synergistic effects may arise either from interactions among phytochemicals within the same extract or from combinations with conventional drugs.

For PG, previous studies have shown that juice or crude extracts may exert stronger inhibitory effects than isolated purified compounds, suggesting synergistic and/or additive interactions among phytochemicals such as proanthocyanidins, anthocyanins, and flavonoid glycosides [168]. In the antiparasitic context, as summarized throughout this review, most reported activities are associated with crude or semipurified extracts (Table 1), indicating that complex phytochemical mixtures may be relevant for efficacy. The complementary actions of these metabolites may explain such outcomes. For example, terpenoids can disrupt membranes, induce oxidative stress, and alter permeability, thereby facilitating the entry of other aromatic molecules that further aggravate membrane damage, interfere with essential parasite functions, and promote cell death [169].

Additive or synergistic interactions with conventional antiparasitic drugs have also been documented, suggesting the possibility of dose reduction and improved therapeutic outcomes. For instance, the combination of diminazene aceturate with a methanolic PG peel extract reduced the effective dose of the drug by approximately 1.6‐fold in babesiosis models, indicating that PG compounds contributed to parasite growth inhibition [116]. Likewise, in cystic echinococcosis animal models, the combination of ABZ with aqueous PG peel extract significantly enhanced inhibition of hydatid cyst growth compared with ABZ alone while also improving liver protection and reducing disease‐associated hepatic damage [170]. In this case, antioxidant and polyphenolic constituents such as punicalagin may complement the antiparasitic mechanism of the standard drug.

Synergistic effects may also involve pharmacokinetic interactions. PG juice has been reported to inhibit cytochrome P450 isoenzymes such as CYP3A4 and CYP2C9, potentially increasing the absorption or bioavailability of drugs metabolized through these pathways, including metronidazole, which is used against several pathogenic protozoa [171]. Although such interactions may be beneficial in some contexts, they should also be considered carefully from a safety perspective.

Clinical translation additionally requires consideration of gastrointestinal bioavailability. Punicalagin and ellagitannins generally exhibit low systemic bioavailability in their native forms after oral administration. However, these compounds undergo extensive metabolism in the gastrointestinal tract: Ellagitannins are hydrolyzed to ellagic acid, which is subsequently transformed by gut microbiota into urolithins (e.g., UA and urolithin B). These metabolites show improved absorption and longer circulation times than the parent compounds, which may contribute substantially to their biological effects [172].

Interestingly, interindividual variability in gut microbiota composition may influence the capacity to generate urolithins, meaning that some individuals may obtain lower systemic exposure to active metabolites. In addition, the relatively slow hydrolysis of ester bonds in ellagitannins can prolong their residence time in the gastrointestinal tract, favoring local activity [173]. This feature may be advantageous for intestinal parasitosis, where direct luminal exposure is desirable, whereas systemic infections may require optimized delivery systems to enhance absorption and tissue distribution.

Nanotechnology‐based strategies may help overcome these limitations. In general, nanoparticles can improve gastrointestinal and vascular permeability, increase selectivity, and optimize the delivery of bioactive compounds to target tissues [174]. PG peel extract has been successfully encapsulated in proniosomal/niosomal vesicles, improving physicochemical stability, encapsulation efficiency, storage performance, and safety while preserving antioxidant activity [175]. Similarly, nanophytosome systems have been explored to enhance the stability, solubility, and biological activity of PG extracts while also enabling controlled release and improved tissue targeting [176, 177].

From a translational perspective, these advanced formulations may be particularly valuable in parasitic diseases, where optimized delivery to intestinal, hepatic, blood, or intracellular infection sites could improve therapeutic efficacy and support the future use of PG‐derived compounds as nutraceutical supplements or adjuvant agents alongside conventional antiparasitic therapies.

## 6. Conclusion

Parasitic diseases remain a major global health concern, particularly in low‐income and resource‐limited regions where they contribute to high morbidity and socioeconomic burdens. Many of these infections are classified as neglected tropical diseases, and the limited development of new therapeutic agents, along with increasing drug resistance, highlights the urgent need for alternative and complementary treatments.

This review demonstrates the potential of *P. granatum* L. (PG) as a natural antiparasitic agent against a wide range of protozoan and helminthic infections. In protozoa, PG‐derived compounds, particularly polyphenols and ellagitannins, impair parasite growth and interfere with essential biological processes, including cytoskeletal integrity, DNA replication, and differentiation. In animal models infected with protozoa, PG administration reduces parasitemia, modulates the immune response, and enhances host recovery by restoring antioxidant balance and tissue architecture.

Similarly, PG exhibits significant anthelmintic properties, extracts of the plant disrupt mating, reduce motility, and induce structural damage, among other effects that lead to parasite death. In cestodes, PG extracts reduce egg output and worm burden or impair neuromuscular activity, resulting in paralysis and death of the parasites. The nematicidal effects of PG have also been reported, where extracts reduce motility and inhibit egg hatching.

The pharmacological activity of PG is attributed to its rich phytochemical composition, which includes polyphenols, tannins, flavonoids, alkaloids, and saponins. These bioactive compounds exert their antiparasitic effects through multiple mechanisms, such as oxidative stress induction, metabolic disruption, tegumental damage, and immune modulation. Given its broad‐spectrum activity, PG represents a promising natural alternative for parasitic disease control. Future studies should focus on standardizing extraction methods, elucidating precise mechanisms of action, and evaluating their efficacy in clinical settings.

## Author Contributions

**Vargas-Villanueva José Roberto:** conceptualization, investigation, writing – original draft. **Gutiérrez-Gutiérrez Filiberto:** investigation, data curation, writing – review and editing, visualization. **Ochoa-Maganda Verónica Yadira:** investigation, writing – review and editing, visualization. **Nery-Flores Sendar Daniel:** conceptualization, data curation, writing – original draft, visualization. **Palomo-Ligas Lissethe:** conceptualization, supervision, data curation, writing – original draft, writing – review and editing.

## Funding

No funding was received for this manuscript.

## Conflicts of Interest

The authors declare no conflicts of interest.

## Data Availability

Data sharing is not applicable to this article as no datasets were generated or analyzed during the current study.
